# Antibody-Mediated Autoimmune Encephalopathies and Immunotherapies

**DOI:** 10.1007/s13311-015-0410-6

**Published:** 2015-12-21

**Authors:** Matteo Gastaldi, Anaïs Thouin, Angela Vincent

**Affiliations:** Nuffield Department of Clinical Neurosciences, University of Oxford, Oxford, UK; University of Pavia, Pavia, Italy; Institute of Neuroscience, Newcastle University, Newcastle upon Tyne, UK

**Keywords:** Autoimmune encephalopathy, Autoantibodies, Immunosuppressive treatment, Voltage-gated potassium channel-complex, *N*-methyl D-aspartate receptor

## Abstract

Over the last 15 years it has become clear that rare but highly recognizable diseases of the central nervous system (CNS), including newly identified forms of limbic encephalitis and other encephalopathies, are likely to be mediated by antibodies (Abs) to CNS proteins. The Abs are directed against membrane receptors and ion channel-associated proteins that are expressed on the surface of neurons in the CNS, such as *N*-methyl D-aspartate receptors and leucine-rich, glioma inactivated 1 protein and contactin-associated protein like 2, that are associated with voltage-gated potassium channels. The diseases are not invariably cancer-related and are therefore different from the classical paraneoplastic neurological diseases that are associated with, but not caused by, Abs to intracellular proteins. Most importantly, the new antibody-associated diseases almost invariably respond to immunotherapies with considerable and sometimes complete recovery, and there is convincing evidence of their pathogenicity in the relatively limited studies performed so far. Treatments include first-line steroids, intravenous immunoglobulins, and plasma exchange, and second-line rituximab and cyclophosphamide, followed in many cases by steroid-sparing agents in the long-term. This review focuses mainly on *N*-methyl D-aspartate receptor- and voltage-gated potassium channel complex-related Abs in adults, the clinical phenotypes, and treatment responses. Pediatric cases are referred to but not reviewed in detail. As there have been very few prospective studies, the conclusions regarding immunotherapies are based on retrospective studies.

**Electronic supplementary material:**

The online version of this article (doi:10.1007/s13311-015-0410-6) contains supplementary material, which is available to authorized users.

## Introduction

Antibody (Ab)-mediated diseases of the central nervous system (CNS) are one of the exciting aspects of clinical neurology. The diseases are associated, and probably caused by, antibodies (Abs) that bind to the surface of neurons. The conditions can be very disabling and patients may need long-term hospitalization, including intensive care, but eventually following immunotherapies, they make a substantial improvement (see [1, 2]).

**Fig. 1:**
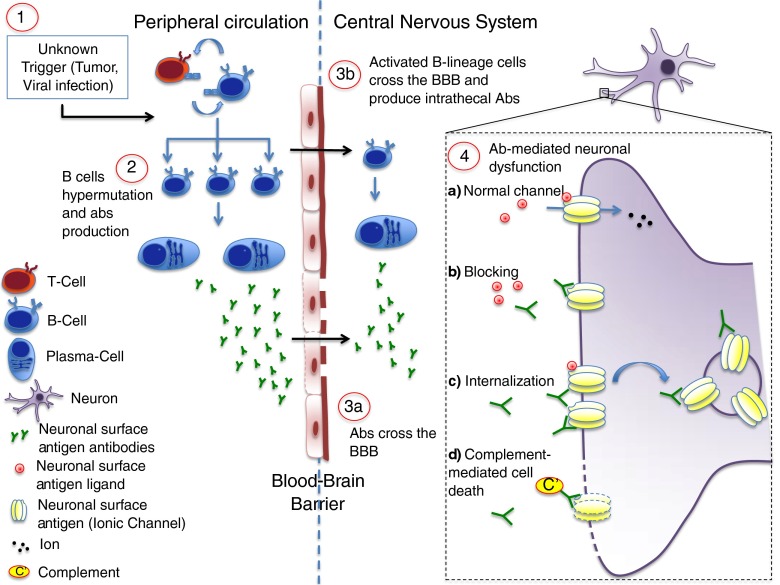
Potential pathogenic mechanisms in antibody (Ab)-mediated autoimmune encephalopathy. The pathogenesis of Ab-mediated encephalopathies is still unclear. Several potential triggers have been proposed as the first determinant of an aberrant activation of the immune system (1). In N-methyl-D-aspartate encephalitis it is well recognized that a tumor (mainly an ovarian teratoma) or a herpetic infection can precede the onset of the disease, but in the majority of cases the trigger remains unknown. In the peripheral circulation B lymphocytes, after interaction with T-helper lymphocytes, become activated and undergo somatic hypermutation and differentiation, starting the auto-Ab production (2). Abs against neuronal surface Ag may subsequently reach the central nervous system by crossing the blood–brain barrier (BBB) at sites of increased permeability (3a). It is also likely that activated B-lineage cells are able to cross the BBB actively and undergo the same differentiation process within the central nervous system, contributing to the intrathecal pool of auto-Abs (3b). When the Abs reach their target, the normal function of the surface Ag (usually a ionic channel; 4a) can be altered by different mechanisms. The Abs may prevent the binding of the channel ligand (blocking; 4b); some Abs cause cross-linking and internalization of receptors and thus depletion from the cell surface (4c); finally, Abs may activate the complement cascade and induce neuronal death (4d)

The distribution of the plasma cells that secrete the Abs and the distribution of IgG throughout the parenchyma of the brain, as well as the roles of other immune effector mechanisms, are largely unexplored, but the Abs are thought to either penetrate a leaky blood–brain barrier (BBB) or to be synthesized mainly within the intrathecal compartment (Fig. 1). This may differ between different Abs.

**Fig. 2:**
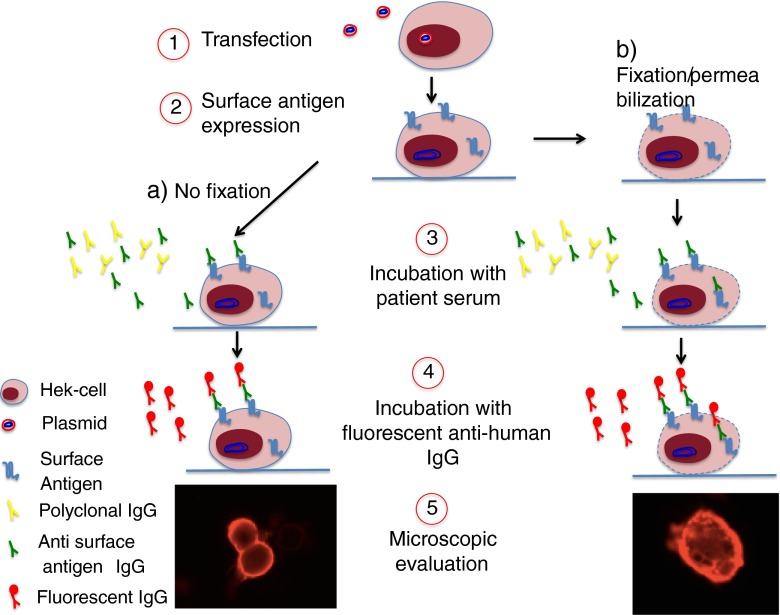
Live and fixed cell-based assay (CBA) for the detection of neuronal surface antigen (Ag) Abs. The CBA is a technique that allows identification of Abs whilst preserving the tertiary structure of the antigen. Live human embryonic kidney (HEK) cells are transfected using plasmids that contain DNA coding for the antigenic target (1); transfected cells express the Ag mainly (but not exclusively) on their surface (2); cells can either be stained live (a), or be fixed and permeabilized (b), and are subsequently incubated with patient serum or cerebrospinal fluid (3). Specific Abs in the serum or cerebrospinal fluid will bind the expressed antigenic target; note that when cells are alive specific Abs are able to bind only antigenic targets expressed on the cell surface (a), whilst when cells are fixed/permeabilized intracellular Ags can be reached (b). Cells are then incubated with a secondary fluorescent Ab that recognizes human IgG (4) and the presence of fluorescent antihuman IgG is detected with a fluorescent microscope (5)

One of the key features of these new conditions with cell surface Abs is that they are diagnosed by use of cell-based assays. These assays use cells that have been engineered to express on their surface one of the potential antigens (Ags). The binding of the patient’s serum or cerebrospinal fluid (CSF) IgG is detected with a fluorescent secondary antihuman IgG, and the cells observed under fluorescence microscopy (Fig. 2). Some laboratories use live cells so that only Abs binding to extracellular domains are detected, while others find that fixed and permeabilized cells are equally suitable. Ab assay kits for the majority of the Agtargets discussed here are now widely available but require fixation for transport, and the sensitivities are not widely established.

Here the Ab-mediated different diseases are described, with an emphasis on what is known about treatment and outcomes.

## *N*-Methyl D-aspartate Receptor

*N*-Methyl D-aspartate receptor (NMDAR) Ab encephalitis is the most common Ab-mediated autoimmune encephalopathy [3], accounting for 4 % of encephalitis of all causes, according to a recent prospective study in England. It was first described in 2005 [4] as a paraneoplastic syndrome associated with ovarian teratomas in young women, and the antigenic target was determined in 2007 [5]. The initial presentation of NMDAR Ab encephalitis appears to evolve from a frequent, but not invariable, prodromal stage, to a clinical presentation with behavioral and personality changes and psychiatric features, including psychosis, cognitive dysfunction, and seizures, often occurring within the first few days of symptom onset. These are followed days to weeks later by subcortical features, such as movement disorder/dyskinesia, autonomic dysfunction, central hypoventilation, and reduced consciousness [6]. Finally the patients recover, often showing previous features in reverse order. CSF and electroencephalography are abnormal in the majority of patients [7, 8], and the findings mirror the clinical course: CSF lymphocytosis and epileptiform changes are a common finding in the first 20 days of the disorder, whereas CSF-specific oligoclonal bands and generalized slowing on electroencephalography tend to appear later [6]. Magnetic resonance imaging findings are abnormal in less than half of patients [7, 8], and when present are usually nonspecific with transient fluid-attenuated inversion recovery or T2 high signal of small cortical areas, basal ganglia, brainstem, cerebellum, or white matter tracts, with some predominance of medial temporal lobe and hippocampal abnormalities [6, 7]. The clinical course is very often severe with most patients requiring admission to intensive care units and disability severity at the peak of disease, reaching a score of 5 on the modified Rankin Scale (mRS) in many [8]. Recovery is often protracted with long hospital admissions [7–9].

NMDAR Abs are usually found in serum and CSF [6, 7, 10]. One study suggested serum testing alone was sufficient for diagnosis, as NMDAR Abs were not detected in the CSF of a few patients with NMDAR Ab encephalitis [6]. However, a study of 250 individuals with NMDAR Ab encephalitis showed that NMDAR Abs, detected using 2 different assays, were present in the CSF of all patients tested, while they were absent from the serum of about 8 % of these patients [10], and the authors recommend testing CSF for diagnosis. The main commercial assay for NMDAR Abs uses this approach but it is not clear whether the sensitivity is the same, and a multicenter study of clinically defined patients needs to be performed.

Absolute titers of NMDAR Ab are higher in serum than CSF [6], but if the IgG concentration in CSF is normalized to that in serum, the relative NMDAR Ab concentration in CSF is almost always higher [7], indicating specific intrathecal production of NMDAR Abs [6, 7]. This is usually taken as evidence of pathogenicity, and of the pathogenic relevance of the CSF Abs, but, in fact, the relative contributions of systemic and intrathecal NMDAR Abs to the disease pathology are difficult to assess and have not been clearly established. Notably, in some diseases [see voltage-gated potassium channel (VGKC)-complex/leucine-rich, glioma inactivated 1 (LGI1) Abs], intrathecal production is much less evident.

NMDAR Ab encephalitis has now been described in all age groups, including infants and the elderly, but it remains chiefly a disease of young women, who comprise 70–100 % of case series [5–8, 11, 12], with a median age at presentation of 21–23 years [6–8]. Initially, most cases were reported to be associated with ovarian teratomas, or rarely other tumors [5, 7], but as case ascertainment has increased, the proportion of tumor cases has dropped to 21–38 % [6, 8], largely because of the increased recognition of NMDAR Ab encephalitis in children and males, in whom tumors are much less common [8, 12]. Indeed, the largest cohort studied to date (577 patients) indicated that 18–41-year-old women were most at risk, and that children of both sexes aged < 12 years, or males, rarely had tumors [8]. In older adults, the prevalence of other tumors, mostly carcinomas, increase again to around 23 % of patients aged 45–84 years [13].

### What is the Evidence for the NMDAR Abs Being Pathogenic?

NMDAR are a subset of ionotropic glutamatergic receptors mediating excitatory neurotransmission. They are ubiquitous in the CNS and, additionally, play key roles in synaptic plasticity and excitotoxicity. The pathogenicity of NMDAR Abs is supported by clinical observations and some *in vitro* and *in vivo* work. First, the early symptoms of NMDAR Ab encephalitis—cognitive deficits, anterograde memory loss, and psychiatric features—bear some resemblance to symptoms caused by NMDAR antagonists in both healthy humans and animal models [14]. Second, CSF NMDAR Ab titers, and to a lesser extent serum titers, correlate broadly with disease course [10]. Indeed, a rapid fall in CSF titers was reported to occur in patients with a monophasic illness and good outcome, whereas the reduction was slower in those with poor outcome [10]. Serum titers drop in successfully treated patients and remain elevated in those with poor outcome or death [6]. Nevertheless, both serum and CSF Abs can be detected in many patients after treatment and recovery [6, 10, 15]. This has management implications with respect to whether and for how long to continue immunotherapies, but in other diseases such as myasthenia gravis, positive Ab titers after good recovery are not uncommon.

NMDAR Abs in patient CSF cross-link surface NMDAR, and thus trigger the internalization of the Ag–Ab complex, leading to selectively reduced NMDAR cluster density on dendrites of cultured neurons and reduced NMDAR-mediated currents [16]. Decreased NMDAR staining intensity in the hippocampi of patients with NMDAR Ab encephalitis has also been observed at autopsy compared with non-NMDAR Ab encephalitis controls. This suggests a specific effect of the Abs in reducing NMDARs similar to those seen *in vitro* [16].

To provide definitive proof of pathogenicity, recapitulation of disease features in experimental animals injected with the Ab is required. *In vivo* evidence of pathogenic effects of NMDAR Abs is still sparse, but a recent study demonstrated memory deficits and some anhedonic behaviors in mice exposed to NMDAR Abs by intracerebroventricular infusion over 14 days, with recovery after cessation of infusion [17]. There was a concomitant reduction in hippocampal NMDAR clusters. Although a limited phenotype was obtained (no seizures, movement disorder, or reduction in consciousness), this finding is still an important demonstration that the effects of the patient Abs *in vitro* do translate to relevant behavioral changes, and justifies the removal of Abs with immunomodulatory treatments. Another study was able to demonstrate increased seizure susceptibility in mice given a single bolus of purified NMDAR Ab IgG with pentylenetetrazol (PTZ) as a proconvulsant [18].

### Treatment

Supportive evidence for pathogenicity is the response to immunomodulatory treatments. There are no prospective trials of different immunomodulatory regimes or agents in NMDAR Ab encephalitis but there are 4 medium-to-large retrospective observational cohorts [6–8, 19], and many small case series and case reports (see Tables 1 and 2). These, mainly retrospective data, can provide useful information on different treatment regimes, as well as the justification for different approaches.

**Table 1:** Patient demographics and immunotherapy response in the 4 largest studies of N-methyl-D-aspartate receptor encephalitis

Description				Tumour and surgical management			Patients treated with ITx		Response to first-line ITx	Second-line ITx used (n, %)	Response to second-line ITx	Relapse rate	Relapse treatment and response
Study	Total patients (*n*)	Age (years), median (range)	Women	*n*	Type (*n*)	Surgically resected (%)	*n* (%)	Percentage tumor surgery	Combination of: IVMP/poCS (75–100 % of those treated) ± IVIg (34–67 %) ± PLEX (~33 %)				
Dalmau et al. [7]	100	23 (5–76)	91	58	Ovarian teratoma (53)	88; earlier tumor resection associated with better outcome; 6 had no ITx	92 (92)		Nonresponders to first-line ITx: 17 (18 %) Overall results (including second-line ITX): full recovery (mRS 0) 47 % (31 % if ITx only) Mild stable deficits (mRS 1–2) 28 % 18 % severe deficits	20 patients (22 %): rituximab (10) cyclophosphamide (9) AZA (1) Alone or combination	76 % responded to cyclophos-phamide and/or rituximab	15 % (1–3 each) 14/15 no tumor or late tumor detection, 0/15 on ITx at relapse	mRS 0–2 in 10/15 with S ± first-line ITx ± rituximab
					Mediastinal teratoma (1)								
					Testicular teratoma (1)								
					SCLC (1)								
					Sex chord stromal tumor (1); neuroendocrine tumor (1)								
Irani et al. [6]	44	22 (2–49)	31 (70)	9	Ovarian teratoma (8)		35 (80)	Outcome NP = P; better with shorter time to oophorectomy	NA Improvement only if receiving ITx within 40 days of onset; trend to better outcome if CS + other ITx	Cyclophosphamide (9 %) rituximab (5 %), AZA (2 %), MMF (2 %), alone or combination	NA	10 % (23 % of NP); 2–4 each, no or limited ITx at relapse	NA
					Hodgkin’s lymphoma (1)								
Titulaer et al. [8]	501	21 (8 months–85 years)	81 %	220/577 38 %	Ovarian teratoma (94 %)	96 % teratomas resected	462 (92)	2 % S alone	53 % response (including S in P group) 96 % mRS 0–2 within 24 months	27 % total (57 % of nonresponders to first-line ITx) Rituximab (20 %) Cyclophosphamide (16 %) AZA/MMF/tacrolimus/MTX (6 %)	67 % mRS 0–2 51 % of nonresponders to first-line ITx evolved to mRS 0–2 without second-line ITx	12 %; NP > > P ITx < < no ITx	NA; ↓ reduced rate if start 2nd line ITx
					Extraovarian teratoma (2 %)								
					4 %: breast/lung/testicular/ovarian/thymic/pan-creatic carcinoma								
Viaccoz et al. [19]	71	25 (18–75)	58 (81)	25 (35 %)	Ovarian teratoma (23) Breast (1) Schwannnoma (1 (male))	N/A	N/A (12/13 male pts)		Not reported, but 5/12 (42 %) treated male patients and 51–60 % female patients received only first-line ITx	Rituximab (42 % of total patients) Cyclophosphamide (10 % of total) AZA/MMF (28 % of total)	Not clearly reported 59 % mRS 0 at 12 months	15.5 %	Not reported

Data are *n* (%) unless otherwise indicated

ITx = immunotherapy; IVMP = intravenous methylprednisolone; IVIg = intravenous immunoglobulin; PLEX = plasma exchange; SCLC = small cell lung carcinoma; NP = nonparaneoplastic; P = paraneoplastic; S = surgery; mRS = modified Rankin Scale; NA = not available; CS = corticosteroids; AZA = azathioprine; MMF = mycophenolate mofetil; MTX = methotrexate

**Table 2:** Immunotherapy response in children and older patients with N-methyl-D-aspartate antibody encephalitis

Patient demographics			Tumor and surgical management		ITx (%)	Response to first-line ITx	Second-line ITx	Response to second-line ITx	Relapse rate (%)	ITx
Study	Female	Median (range) age (years)	Type	Surgery (%)		Combination CS/IVIg/PLEX				
Florance et al. [11]	32 (81)	14 (23 months–18 years)	Ovarian teratoma (31 %)	100	97	77 % improved: full recovery 29 % Substantial improvement 45 % Limited improvement 26 %	23 % rituximab (*n* = 2), cyclophosphamide (*n* = 1) both (*n* = 4)	60 % improved; 40 % slow but progressive improvement	25 (all NP)	NA
Titulaer et al. [8]	177 (74)	NA	Mostly/all ovarian teratomas (details NA)	100 (*n* = 35)	95 (*n* = 168)	49 % responded; 98 % of these reached mRS 0–2 at 24 months	56 of total (32 %) Rituximab (*n* = 42) Cyclophosphamide (*n* = 29) Other (*n* = 11)	Overall at 24 months: 86 % mRS 0–2 (81 % of those receiving second-line ITx *vs* 65 % of those who failed first-line ITx but received no further treatment)	NA	NA
Armangue et al. [12]	20 (70)	13 (8 months–18 years)	Ovarian teratoma (*n* = 1), Follicular cyst (*n* = 1)	100 (*n* = 2/2)	20	60 % improved	Rituximab ± cyclophosphamide (*n* = 7) +MMF (*n* = 2)	100 % improved; overall (firs ± second line): 60 % full recovery 25 % mild disability 10 % severe disability 5 % death (*n* = 1)	0	
Wright et al. [20]	31 (74)	8 (22 months–17 years)	Ovarian teratoma (*n* = 1)	100	100	Full recovery 50 % Partial recovery 30 % None 20 % Suggestion that regime with PLEX superior	32 % of patients. Rituximab (*n* = 3) Cyclophosphamide (*n* = 3) Both (*n* = 3) MMF (*n* = 1)	Full recovery 80 % Partial recovery 20 % Long-term outcome not related to use of second-line ITx; instead, good outcome more likely in early diagnosis	23 % 6/7 prior first-line ITx	First-line ITx in all + second-line ITx in 4/7 prevented further relapses
Titulaer et al. [13]	31 (55)	52 (45–84)	Ovarian teratoma (*n* = 1), Thymic cancer (*n* = 1), ovarian cancer (*n* = 1), breast cancer (*n* = 2), lung cancer (*n* = 2)	71	91	45 % improved	44 % of nonresponders to first-line ITx Cyclophosphamide and/or rituximab	Good response 6/7; Overall 60 % mRS 0–2		

ITx = immunotherapy; IVIg = intravenous immunoglobulin; PLEX = plasma exchange; NP = nonparaneoplastic; NA = not available; mRS = modified Rankin Scale; MMF = mycophenolate mofetil

### Tumor Resection

All teratomas examined histologically were found to contain neurons that expressed NMDAR, and were able to bind patient Abs. When present, resection of the tumor is important for recovery [6–8] In most patients, a combination of surgery and first-line immunotherapy [corticosteroids, intravenous immunoglobulin (IVIg) and plasma exchange (PLEX)] is required [6, 9, 11, 21, 22], and results in improvement in up to 80 % of patients [9]. This highlights the importance of performing a thorough tumor search early in the course of any autoimmune encephalitis, especially in females aged 12–45 years, and black and Asian women, in whom teratomas are found in nearly half the cases of NMDAR Ab encephalitis [8].

### First-line Immunotherapy

Whereas tumor resection is important in those with an ovarian teratoma, first-line immunotherapy alone (corticosteroids, IVIg, PLEX alone or in combination) results in satisfactory clinical improvement in approximately half the patients with nonparaneoplastic NMDAR Ab encephalitis [8, 9, 12, 13]. Generally, the efficacy of individual first-line treatments cannot be distinguished in these studies, as the clinicians chose the treatments or combination of treatments, based on availability, perceived risk, and other factors, and there were no comparison regimes. One case series of 9 patients found that, in those patients who ultimately had a good outcome, PLEX had been started early and had been part of the initial therapy (alone or with corticosteroids) [23]. However, there was such heterogeneity in the patients, both in terms of presence of teratoma and timing and types of treatment, that disentangling the effect of PLEX itself seems ambitious. There were few adverse events associated with the use of PLEX. Perhaps a more appropriate conclusion is that early immunomodulatory treatment may be better than late treatment and that PLEX appears safe in patients with NMDAR Ab encephalitis, including those with autonomic dysfunction.

The case for early treatment has been made in several studies [6, 8, 12, 15, 24–26]: a small case series reported that 4 of 5 children treated with combinations of first-line immunotherapy within 6 days of symptom onset recovered fully with no relapses [24]. The strongest evidence in favor of early treatment comes from the largest observational cohort published, which demonstrated that early treatment, the lack of need for intensive care admission, and maximum mRS score of ≤ 3 were independently associated with good outcome [8]. In that study, about half the patients who received first-line immunotherapy improved within 4 weeks of treatment, and 97 % of these patients went on to have a good outcome (mRS 0–2) at 24 months of follow-up.

Most studies have used the modified mRS to measure outcome. Even in those patients classified as having good outcome (mRS 0–2), deficits in executive function and memory are common and are more severe in those with delayed treatment [15]. This would suggest that the initial part of the illness may be critical in terms of neuronal damage and long-term sequelae. Pathological studies have not demonstrated significant complement deposition and cytotoxicity but have generally been conducted late in the disease and after some immunotherapy [27, 28]. Perhaps complement or cell-mediated toxicity might occur in the early stages of disease if left untreated, setting the path for an incomplete recovery in such patients.

### Second-line Immunotherapy

The most commonly used second-line immunotherapies are rituximab and cyclophosphamide alone or in combination. The use of other agents, such as methotrexate (MTX), azathioprine (AZA), and mycophenolate mofetil (MMF), has not been reported widely enough to permit conclusions to be drawn about their effectiveness, and are more relevant to long-term maintenance. Second-line immunotherapy is required more often in patients with nonparaneoplastic NMDAR Ab encephalitis [8], and substantial improvements are seen in around 75 % of patients treated [7–9, 11–13, 23, 25]. In the largest observational cohort [8], the use of second-line immunotherapy was also identified as a predictive factor for good outcome. Relapses are also less likely in those treated with second-line immunotherapy [8, 29].

At present, it is not possible to determine which drug, combination of drugs, or regime is most effective. The most commonly used regimes are weekly rituximab infusion for 4 weeks and monthly cyclophosphamide infusion for up to 6 months. Few serious adverse effects have been observed in adults. By contrast, a 7.6 % rate of infection (2.8 % causing disability or death) and 2.0 % rate of anaphylactic infusion reactions were reported in 1 study of children with NMDAR Ab encephalitis and other inflammatory and autoimmune CNS disorders [30]. Anaphylactic reactions or infections were reported in 2 % of patients with NDMAR Ab encephalitis treated with rituximab, and infection or severe lymphopenia causing discontinuation of treatment in 2.5 % of patients receiving cyclophosphamide [8]. Progressive multifocal leukoencephalopathy has not been reported in the NMDAR Ab encephalitis literature in the > 130 patients treated with rituximab alone or in combination with other immunomodulatory agents [3, 7, 8, 11, 12, 23]. Cyclophosphamide treatment did not lead to any irreversible adverse effects in any of the cohorts included, but, in view of the age distribution of NMDAR Ab encephalitis, it is important to bear in mind the risk of gonadal failure and infertility, as well as malignancy, in patients receiving large cumulative doses of the drug [25].

Cyclophophamide, but not rituximab, is able to cross the BBB, which is not overall disrupted in patients with NMDAR Ab encephalitis [7]. While the effect of rituximab may be to reduce the supply of B-cell precursors to CNS plasmablasts, and alter the resultant inflammatory environment in the CNS [31], cyclophosphamide could have a direct effect on intrathecal Ab synthesis.

Few studies have systematically studied the effect of immunotherapy on Ab levels. CSF titers mirrored the clinical course more closely than serum levels in a study of 10 patients [10], but the effect of first- and second-line treatments were not differentiated. Case reports occasionally describe reduction [6, 22, 26, 32] or eradication [33, 34] of NMDAR Abs in serum following first-line immunotherapy in patients with good outcome, with concomitant decrease in CSF titers [6, 22, 26, 32–34]. Reductions in CSF titers with second-line treatments have also been demonstrated [31, 35, 36], but for obvious practical reasons CSF titers are not widely available in patients who have improved. Successful aggressive immunosuppression with intrathecal MTX and intravenous (IV) alemtuzumab was reported in 4 children [37, 38], 2 of whom were refractory to prolonged immunotherapy, including cyclophosphamide and/or rituximab. In 2 cases, intrathecal therapy with MTX was associated with a reduction in CSF Ab levels, which was followed by a much slower and inconsistent drop in serum levels [37]. Intrathecal MTX treatment carries the risk of long-term cognitive impairment, which may contribute to the sequelae of NMDAR Ab encephalitis, and should therefore only be considered if lack of response to more commonly used agents is well established.

One remarkable feature of NMDAR Ab encephalitis is the prolonged recovery, with progressive improvements in cognitive domains noted for months and even years following the end of treatment [13, 32]. Although this may be related to the recovery of physiological NMDAR homeostasis as the Ab levels decline, the role of persistent NMDAR Abs in treated patients is not yet clear. Prospective studies are needed to demonstrate the relationship between CSF and serum titers, stage of disease, treatment responses, and final outcomes, including detailed cognitive and mental state.

### Relapses

The relapse rate in NMDAR Ab encephalitis is reported to be 12–25 % [6–8, 11, 29], and relapses may occur months to several years after the initial episode. They are often less severe and may be mono- or pauci-symptomatic. In children, atypical presentations such as cerebellar ataxia or brainstem signs have been described [29]. The majority of relapses occur in patients who do not have a tumor associated with NMDAR Ab encephalitis, those who received no or limited treatment for the initial episode [6–8, 11, 29], and those not exposed to second-line agents [29]. Second-line immunotherapy also appears to prevent further relapses in those with a multirelapse disease course [8]. At present, there is no clinical or paraclinical predictive marker for relapses. Although alterations in CSF Ab titers relate well to clinical changes [10], it is impractical and perhaps unsafe to conduct CSF analysis for predictive purposes in well patients. Changes in serum Ab titers were not well correlated with relapses [10]. The effects of long-term immunosuppression with oral agents such as AZA or MMF on relapse rate is currently unknown.

### NMDAR Ab Encephalitis Following Herpes Simplex Virus Encephalitis

Some children and adults with herpes simplex virus (HSV) encephalitis and subsequent relapses characterized by choreoathetosis in children and behavioral/psychiatric features in adults [39–41], were found to have NMDAR Abs without HSV reactivation. They improved with immunotherapy [39–42]. The absence of HSV DNA in the CSF at the time of relapse could suggest that further antiviral treatment is unnecessary, but in most cases reported, aciclovir was given by default.

## Voltage-gated Potassium Channel Complex

The voltage-gated potassium channel (VGKC) complex Abs are the second most commonly identified Abs in CNS Ab-mediated diseases, but the clinical spectrum and treatment responses are very different from those of the NMDAR Ab encephalitis. The clinical spectrum includes both central and peripheral nervous system disorders.

There are many families and subtypes of VGKCs but the Abs referred to here are those that immunoprecipitate Kv1.1, 1.2, and 1.6 subtypes from mammalian brain tissue extracts. The VGKC complex is composed of Kv1 subunits and other proteins that are tightly complexed with the Kv1 subunits in the nerve membrane. They are widely expressed in the nervous system, particularly at the juxtaparanodes of the nodes of Ranvier, at peripheral motor nerve, and perhaps sensory terminals, and in central synapses. Because opening of the VGKC following each action potential leads to repolarization of the membrane, VGKCs regulate neuronal activity throughout the nervous system.

VGKC complex Abs were first described in association with neuromytonia (NMT) or Isaac’s syndrome. This is characterized by peripheral nerve excitability that manifests with spontaneous muscle contraction, stiffness, sometimes impaired muscle relaxation [43], and a specific electromyography pattern of burst of random discharges at high frequency in the muscle fibers [44]. It is caused by hyperexcitability of the motor nerves leading to repetitive and spontaneous activity in the muscles. VGKC complex Abs were found in around 40 % of patients with NMT and then more frequently and at higher levels in patients with NMT associated with dysautonomia and CNS disturbance including insomnia and limbic dysfunction, which is usually called Morvan’s syndrome (MoS) [45, 46], and in a form of nonparaneoplastic limbic encephalitis (LE).

Although these Abs were initially identified by immunoprecipitation of radioactive dendrotoxin-labeled VGKCs in digitonin-solubilized mammalian brain homogenates, further evidence indicated that they bind to protein components of the VGKC complex rather than to the VGKC itself; their identification requires Ag-specific cell-based assays [47, 48]. The main targets for the Abs are LGI1, typically associated with LE, and a specific focal epilepsy, faciobrachial dystonic seizures (FBDS), and contactin-associated protein like 2 (CASPR2), associated with a broader spectrum of central and peripheral nervous system disorders such as LE, NMT, or a combination of the two (MoS). A third antigen, contactin 2, has been identified, usually in patients with concomitant anti-LGI1 or anti-CASPR2 Abs and with no specific phenotype, suggesting an unclear clinical relevance.

In some patients VGKC Abs are detected by radioimmunoassay, in the absence of LGI1, CASPR2, or contactin 2 Ab specificity. In general, high titers (usually > 400 pM) are more frequently associated with defined clinical phenotypes (mainly LE, but also peripheral nerve hyperexcitability (PNH), seizures, or MoS), whereas low titers of Abs can be found in patients with either or both CNS and PNS syndromes but are also detected in patients without a clear immune-mediated phenotype [49–51]. It is possible that, in these cases, the Abs bind to intracellular targets on the solubilized VGKC complex [52].

### VGKC Complex Ab LE

The most important association of VGKC complex Abs is with LE. LE is defined as the combination of seizures, memory impairment, behavioral changes, and sleep disturbances, and was previously thought to be only paraneoplastic with Abs directed against intracellular cerebellar Ags [53]. LE with Abs against the VGKC complex was the first form of CNS disease shown to be potentially reversible and to respond reliably more quickly to immunotherapies [54]. Abs to LGI1 are the most common finding and there is a good correlation between Ab titers and clinical syndrome, suggesting that they are pathogenic [55]. Immunopathology studies of patients with VGKC complex LE show, differently from NMDAR Ab encephalitis, perivascular lymphocytic infiltration and neuronal loss predominantly in the hippocampus and the amygdala [56]. The presence of immunoglobulins and complement deposition on neurons suggests that complement-mediated neuronal death has—surprisingly, as a proportion of the Abs are IgG4, which does not activate complement—a prominent role in the disease compared to T-cell-mediated cytotoxicity [57]. There are few published *in vitro* or *in vivo* data to support the pathogenicity of the CNS-directed Abs, but purified IgG from a VGKC complex/LGI1 Ab serum increased cell excitability in rat hippocampal slices within a 2-h incubation period [58], suggesting that the Abs have epileptogenic properties. A more detailed and comprehensive paper showed that the LGI1 Abs disrupted binding of LGI1 to its partners ADAM22 and ADAM23 in cultured neurons with effects on the expression of α-amino-3-hydroxy-5-methyl-4-isoxazolepropionic acid receptors (AMPAR; excitotoxic), suggesting that both presynaptic VGKCs and AMPAR function might be reduced in these patients [59]. As AMPARs are excitatory and VGKCs have an inhibitory effect on neuronal excitability the consequences of the changes are difficult to predict.

### Treatment of LE

A review of papers with relevant information regarding VGKC complex Ab-associated disease management and outcome is summarized in Supplementary Table 1. Most studies are retrospective and there is a lack of standardized methods to assess clinical status after treatment. Moreover, information on treatment can be confused by the variability of the inclusion criteria of studies that can be based either on clinical presentation or on the target of the detected Ab.

Patients with VGKC-related disease can rarely improve spontaneously or with anticonvulsant treatment alone [60], but in the majority of cases immunosuppression is required for sustained clinical improvement, especially in patients presenting with LE [54, 61]. Marked improvement of cognitive function and seizure control can occur shortly after administration of immunotherapy, and the resolution of clinical symptoms often correlates well with Ab levels [62]. As previously stated, the main Ag in LE is LGI1, but the disease can occur also in association with CASPR2 Abs, also with a good response to immunosuppression [63].

Different treatment options in the acute phase involve oral or IV steroids, PLEX, and IVIg, but no randomized clinical trial has established a first-line drug or optimal treatment duration. Generally, 2 different approaches could be suggested: 1) starting with 1 first-line therapy (e.g., corticosteroids), eventually switching to a combination therapy if incomplete response or relapses manifest; or 2) starting with a combined treatment (e.g., steroids plus IVIg). Published data suggest that the latest approach could be preferable in terms of cognitive improvement, and is also associated with a higher rate of treatment-related complications [64]. Vincent et al. [62] reported that although some patients presented a dramatic response to PLEX or IVIg, most presented a consistent improvement only after a few weeks of oral steroids. Thieben et al. [61], after treating patients with VGKC Ab and LE with high dose intravenous 6-methyl prednisolone (IVMP), found that improvement was dramatic if patients were symptomatic for ≤ 2 months. If clinical symptoms were present for > 9 months recovery was often incomplete, suggesting that early treatment could be beneficial in LE.

In an open-label prospective trial, 9 of 9 patients with VGKC complex Ab-related LE showed clinical improvement after treatment with a combination of PLEX, IVIg, and IVMP followed by maintenance with oral steroids, further supporting the notion that a combination therapy and prolonged immunosuppression could be more effective in determining a long-term remission [65].

Although compared with other Ab-mediated encephalopathies, such as NMDAR Ab encephalitis, relapses are uncommon in VGKC Ab-associated disease, recurrence of symptoms, sometimes as a result of suspension or noncompliance of treatment, has been described. In these cases, symptom recurrence is often associated with persisting serum Abs or increasing titers [66]; these findings could therefore be helpful in determining the opportunity of immunotherapy suspension.

In refractory cases, additional immunosuppression could then be necessary. PLEX has shown improvement lasting for several months in some patients either in combination or as the only treatment, but is rarely considered as a long-term option [67]. Different immunosuppressive drugs such as AZA, MMF, tacrolimus, and rituximab have been used as second-line or “steroid-sparing” agents on a limited number of patients, usually with more severe clinical presentation, and the results are highly variable. Rituximab has been used in a case series of 5 patients [68]. Despite the fact that all patients were treated quite late in the history of the disease (at least 1 year after the first symptom), 2 of them showed a beneficial effect, with reduction of seizures and cognitive improvement, suggesting that the drug could be a valid option in refractory patients. Even though the outcome of the disease is generally good, many patients show residual memory deficits after the resolution of the acute phase [69]. Whether a more aggressive approach involving the use of an immunosuppressant like rituximab in the early stages of the disease could prevent cognitive impairment is intriguing, and needs to be explored.

Finally, the presence of a tumor is a rare event in patients with VGKC Abs, especially if presenting as LE with LGI1 Ab, but can be more common in patients with MoS or NMT with CASPR2 Abs. In such patients, a thymoma is common, and the outcome is influenced by the evolution of the tumor [46, 48].

### FBDS

In around 20–40 % of patients with LGI1 Abs a specific seizure type can precede the occurrence of full-blown LE [70]. These events have been described as “tonic seizures” [71], or FBDS [66], and consist of brief and very frequent involuntary movement with dystonic features involving mainly the arm, the ipsilateral side of the face, and, less frequently, the leg. The response to routinely used anticonvulsant drugs is usually poor, but a dramatic reduction or complete resolution of the FBDS can be obtained with oral steroids [72]. Irani et al. [66] described a prospective cohort of 10 patients with FBDS, where the development of cognitive impairment was only present in patients who did not receive immunotherapy, suggesting that early treatment can result in a better recovery and sometimes prevent progression to encephalopathy.

### Dipeptidyl-peptidase-like Protein-6

Dipeptidyl-peptidase-like protein-6 is a protein associated with another VGKC, Kv4.2, that is responsible for regulating firing rates of action potentials in dendrites in the central and peripheral nervous system. Abs to dipeptidyl-peptidase-like protein-6 were initially identified in patients with a form of LE associated with gastrointestinal dysmotility (due to the involvement of the myoenteric plexus), sleep disturbances, cognitive and psychiatric manifestations, and dysautonomic features [73], and subsequently in a disease presenting with hyperekplexia, trunk rigidity, and cerebellar ataxia [74]. Overall, most patients seem to respond to immunosuppression, irrespective of the treatment strategy chosen. In a recent review of 20 cases, 8 of 12 patients treated with immunotherapy improved, but clinical data were mostly collected retrospectively [75].

## Other Abs

### Antiglutamic Acid Decarboxylase

Abs to glutamic acid decarboxylase (GAD), the rate-limiting enzyme in the synthesis of the inhibitory neurotransmitter γ-aminobutyric acid (GABA), have been reported in a number of different neurological syndromes, including stiff person syndrome, cerebellar ataxia, LE, and epilepsy, as well as in individual patients with isolated neurological symptoms. The incidence of GAD Ab-associated LE is unknown as the literature consists mostly of case reports [76–82], and retrospective studies reporting 9 and 16 patients, respectively [83–85]. GAD Ab-associated encephalitis is often nonparaneoplastic and presents as a more typical limbic syndrome than many of the autoimmune encephalitides described above, with subacute evolution of memory impairment and temporal lobe seizures, which are mostly resistant to treatment with antiepileptic drugs. Brain imaging usually reveals uni- or bilateral medial temporal lobe hyperintensity on T2/fluid-attenuated inversion recovery sequences. The role played by the GAD Abs is unclear. GAD Abs are found in both serum and CSF of patients with LE, and CSF-specific oligoclonal bands and intrathecal synthesis of GAD Abs are most often present [76, 79, 84]. However, Ab access to its target is not straightforward as GAD is an intracellular enzyme. Also, the range of syndromes associated with GAD Abs would suggest that the Abs can have different and nonoverlapping effects within the CNS. It seems more likely that GAD Abs may be markers of the immune-mediated process in LE: coexistence with GABA_B_ receptor (GABA_B_R) Abs has been described [86], raising the possibility that other cell surface Abs may be important, and biopsies have demonstrated marked neuronal loss and T-cell infiltrates, with no IgG deposition [57, 79, 84]. Of note, a recent study [87] found GAD Ab-associated LE to be 10 times more likely to be paraneoplastic than GAD Ab-associated stiff person syndrome or cerebellar ataxia, but GAD Abs are quite frequently found in patients with other, more pathogenic, Abs. Indeed, older age and the presence of additional Abs against neuronal cell-surface Ags (especially GABA_B_R) were markers of the paraneoplastic nature of the syndrome.

The effects of immunotherapy are variable and often disappointing. Combinations of corticosteroids, IVIg, and PLEX have been found to be effective in a number of case reports [57, 77, 79, 82], but treatment often had to be continued for several months [79, 82], or be augmented with a second-line agent, most commonly MMF [76, 82]. Outcome measures were generally not described and seizures often persisted [82]. The two case series reported only very modest improvements with immunotherapy [83, 84]. Seizure frequency reduced somewhat, although no patients became seizure-free [84], and detailed neuropsychological assessment revealed that although executive function improved, this was not matched by an improvement in memory [83]. Ab levels appeared to decrease with treatment but it is important to note that in all cases they remained significantly elevated.

Overall, there is little doubt that an immune-mediated process is taking place in the medial temporal lobes of patients with LE and GAD Abs, and many patients reported thus far were treated after significant delays. A potential beneficial effect of early immunotherapy cannot be ruled out.

### AMPAR

AMPAR are a subgroup of ionotropic glutamate receptor mainly present in excitatory synapses of the CNS. Abs against the extracellular domains of AMPA subunits GluR1 and GluR2 were associated originally with a particularly aggressive form of LE, often accompanied by the presence of a tumor [88]. The largest case series reported describes 22 patients and shows that the clinical spectrum can include, in addition to LE, psychosis and multifocal encephalopathy. Administration of first-line treatment (steroids or IVIg) and, when appropriate, tumor removal, often lead to a complete or partial remission of the symptoms. The patients can relapse, but a more aggressive course of treatment involving rituximab and/or cyclophosphamyde seems to be associated with a monophasic disease [89].

### GABA_B_R

GABA_B_R is a protein widely distributed in the brain and located both pre- and postsynaptically. Genetic alterations of the receptor are associated with epilepsy and cognitive impairment, and Abs against the B1 subunit are found in patients with LE and, rarely, ataxia, 50 % of whom will have a small-cell lung carcinoma [86]. The administration of immunotherapy, in association with chemotherapy or tumor removal, is accompanied by a prompt improvement and, in a percentage of cases, by a complete recovery [90]. Conversely, patients that did not receive immunotherapy had a bad prognosis, and death occurred in a high percentage of cases within months from the onset of the neurological disease. Some patients have a poor outcome despite sustained immunosuppression, but that is often related to tumor progression or associated with the presence of Abs directed against intracellular Ags such as GAD Abs or amphyphysin Abs, which can reflect the involvement of an additional cytotoxic T-cell mechanism in the progression of the disease [91].

More recently, a novel Ab against GABA_A_ receptor has been described [92]. Pedrol-Petit et al. [92] reported 18 patients with Abs directed against the α1/β3 subunit of the receptor, 6 with high titers in serum and CSF and a definite clinical picture, and 12 with low titers only on serum and variable syndromes, often in association with other Abs. Among the 6 patients with CSF Abs, only 1 had substantial recovery with antiepileptic drugs alone, which had to be maintained for a long time to avoid recurrence. The remaining 5 patients received different immunotherapy regimens (either steroids alone or in combination with IVIg, PLEX, cyclophosphamide or rituximab), with a consistent recovery in 3 of them, whilst 2 died because of septic complications.

Another paper described 40 patients with Abs of the IgG and IgM subclasses targeting the α1 and γ2 subunits of the GABA_A_ receptor [93]. Immunosuppressive treatment was administered prospectively only to 1 patient, whose catatonia and frontal dysfunction improved with a fall in Ab titres.

### Glycine Receptor

Abs directed against the α1 subunit of the glycine receptor (GlyR) have been described in patients with variants of stiff person syndrome usually identified as progressive encephalomyelitis, rigidity, and myoclonus, which is characterized by the association of rigidity, stimulus-sensitive spasms, myoclonus, hyperkeplexia, autonomic disturbances, and brainstem disorders [94]. In the only prospective case series, patients treated with immunotherapy showed consistent improvement, sometimes with complete resolution of clinical symptoms. Therapeutic approaches were variable but typically involved a combination of IVMP, PLEX, and IVIg followed by oral prednisolone. Nevertheless, 6/45 patients relapsed and required prolonged immunosuppression with MMF, AZA, or cyclophosphamide, which, at the time of publication, were effective in preventing further relapses. Recently, GlyR Abs have also been identified in association with isolated optic neuritis [95], and they represented the most frequent Ab recognized in patients with focal adult epilepsy of unknown cause [96]. However, in these studies the Abs were also found in a consistent number of patients in the control group, the titers did not correlate clearly with disease presentation or drug administration, and the response to immunosuppression was highly variable. The role of GlyR Abs in such conditions may not be clinically helpful, although it could reflect the presence of autoimmune mechanisms.

### IgLON5

In 2014, a novel syndrome with sleep disorders (parasomnia and breathing dysfunction), gait instability, and brainstem symptoms was described in 8 patients in association with surface Abs to the neuronal cell adhesion protein IgLON5 [97]. Neuropathological investigations in 2 patients identified tau aggregates in the tegmentum of the brainstem and in the hypothalamus that could not be classified within any known tauopathy, suggesting a possible neurodegenerative etiology of the disease. Moreover, despite immunosuppressive treatments including steroids, IVIg, cyclophosphamide, and rituximab, only 1 patient showed some improvement. Whether the Abs are a primary or secondary element in the disease development needs to be clarified.

### Dopamine 2

Dale et al. [98], investigating the role of surface Abs in suspected autoimmune movement disorders, identified Abs to the extracellular domain of the dopamine receptor 2 in 12/17 patients with basal ganglia encephalitis compared with 0/67 controls [98]. In addition, dopamine receptor 2 Abs were found in a small number of patients with Tourette’s syndrome and Syndenham’s chorea. Most patients in the study were identified retrospectively and were given no immunotherapy, presenting at the end of follow-up with persistent neurological deficits. Interestingly, patients identified after the study who received immunosuppression showed marked clinical improvement and a reduction in Ab titers (Dale et al., personal communication), suggesting a consistent role of the Ab in disease progression. Further studies are required to confirm these data and help define an optimal treatment course.

### Metabotropic Glutamate Receptor

Subacute cerebellar ataxia associated with metabotropic glutamate receptor type 1 Abs (mGluR1) is a rare clinical entity, so far described in 3 reported patients [99, 100]. Paraneoplastic cerebellar degeneration is often associated with Abs directed against cytoplasmic or nuclear Ags that are important for the diagnosis but unlikely to be directly responsible for neuronal damage. By contrast, mGluR1 Abs are directed at a surface Ag on perisynaptic dendritic spines of Purkinje cells and, in one study, shown to be pathogenic [99].

More recently, Abs directed to the mGluR5 Abs were identified in 2 patients with limbic encephalopathy and Hodgkin lymphoma, a combination known as Ophelia syndrome [101]. In both cases prompt tumor treatment (excision or chemotherapy) was performed, and 1 patient also received steroids, resulting in complete regression of the symptoms.

## Conclusions and Questions for the Future

Despite the interest in identifying the autoimmune forms of encephalitis described here, and the introduction of auto-Ab testing in centeRs around the world, the low prevalence of these disorders and their relatively recent discovery has limited comparison of different treatment protocols. Prospective studies on outcomes in patients with defined clinical and Ab subtypes are required in the future, to define optimal therapeutic strategies.

Despite these limitations, some general suggestions can be made. First, many patients have improved considerably and returned to a near-normal life. Although in these conditions there is still much to know (possibility of wider clinical phenotypes, natural history, and long-term outcome, efficacy of different immunotherapies), the relative safety of immune active drugs, especially in the short-term, provides a rationale for treatment. Even when the clinical picture is not life-threatening, immunosuppression can be considered in the syndromes associated with the NMDAR or VGKC Abs, and disease evolution will be the main element guiding further decisions (e.g., treatment escalation or tapering). Although Ab titers in isolation are not sufficient to guide treatment choice, carefully analyzed serial samples can be helpful in deciding whether treatments have been sufficient and in determining when to start tapering the drugs.

**Fig. 3:**
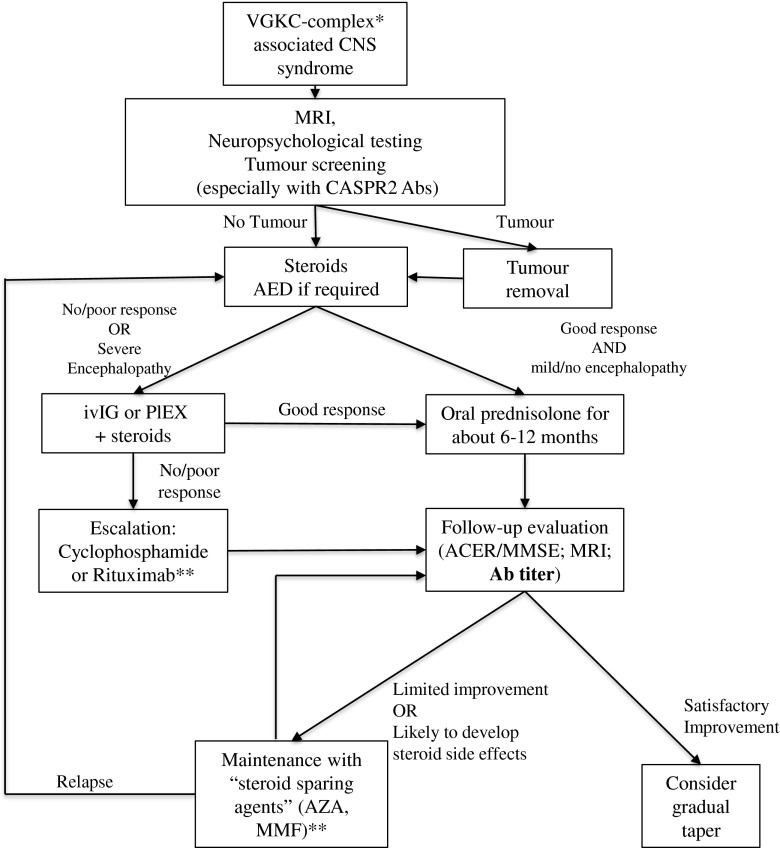
Management of voltage-gated potassium channel (VGKC) complex-associated disease. The algorithm describes a management approach to patients with central nervous system (CNS) syndromes and the presence in serum or cerebrospinal fluid of Abs directed against the VGKC complex, and it is intended as a general indication. Single patients could need a tailored approach based on the clinical phenotype *Mainly leucine-rich glioma inactivated 1 and contactin-associated protein like 2 (CASPR2) **The efficacy of different immunosuppressants has never been tested systematically in autoimmune encephalitis, and previous evidence suggests, for example, a better efficacy of mycophenolate mofetil (MMF) than cyclophosphamide in other autoimmune diseases [102]. Hence, the division between “escalating” and “steroid-sparing” drugs is not clear-cut and different strategies could be applied in different clinical settings MRI = magnetic resonance imaging; AED = antiepileptic drugs; IVIg = intravenous immunoglobulin; PLEX = plasma exchange; ACER = Addenbrooke’s Cognitive Examination Revised; MMSE = Mini-mental State Examination; AZA = azathioprine

Somewhat different approaches to treatment are required for the different diseases. NMDAR Ab encephalitis is characterized by long-lasting Abs in patients and often a prolonged disease that can relapse. Moreover, some key feature of the disease (such as hypoventilation) can be fatal. Thus, a relatively aggressive treatment approach, with extensive use of immunosuppressive drugs such as rituximab and cyclophosphamide for several months, may be justified, even in combination. However, the LGI1-associated disease spectrum spans from patients with FBDS only to patients with severe encephalopathies, and less persistent aggressive treatment is often appropriate; PLEX or IVIg with oral steroids is successful in many patients, although some require second-line therapies. Relapses are not common, and steroid tapering can usually be attempted within a year. An algorithm suggesting a general approach in patients with VGKC associated disease is presented in Fig. 3.

In contrast to NMDAR Abs, which are mainly IgG1, LGI1 Abs are mainly IgG4, a subclass that accounts for < 5 % of total IgG and is traditionally produced as an anti-inflammatory mechanism after prolonged exposure to Ags [103]. However, IgG4 Abs are now associated with several diseases involving the nervous system (e.g., myasthenia with muscle-specific kinase Abs) and other organs (pemphigus, Goodpasture’s syndrome, membranous nephropathy, thrombotic thrombocytopenic purpura) [104]. As these Abs are not able to activate complement, the likely pathogenic mechanism is Ab binding to essential functional sites on the Ag [105]. Interestingly, in IgG4-related disease plasma cells that produce IgG4 are usually short lived [106], and rituximab has been shown to be very effective in muscle-specific kinase Ab myasthenia. Further studies will need to investigate whether the IgG subtype involved in the disease could have an impact on the treatment strategy.

Finally, future studies will need to focus on increasing the knowledge on pathophysiology of different Ab-mediated encephalitides, leading to more pharmacological approaches to use while immunotherapies take effect, and to develop more targeted therapeutic strategies. For instance, new therapeutic options should try to tackle intrathecal synthesis of specific Abs in the CNS.

Rituximab is effective in depleting CD20+ cells, but in patients treated intravenously CSF concentration of the drug is only 0.2 % compared with serum [107, 108]. Therefore, intrathecal drug administration could be more effective in reducing CNS NMDAR Ab production. Natalizumab, a monoclonal Ab against α-4 integrin that affects trafficking of T and B cells across the BBB [109], has been successful in multiple sclerosis in altering the oligoclonal band pattern and the intrathecal synthesis. This drug could prevent B-cell trafficking into the brain in NMDAR Ab encephalitis.

None of these drugs are without potential side effects but the severity of the disease in some patients justifies further consideration of these approaches.

## Electronic supplementary material

Below is the link to the electronic supplementary material.

Supplementary Table 1 Treatments in representative studies of patients with VGKC associated disease (PDF 612 kb)
